# Probing Reactivity with External Forces: The Case of Nitroacetamides in Water

**DOI:** 10.3390/molecules29010009

**Published:** 2023-12-19

**Authors:** Giovanni La Penna, Fabrizio Machetti

**Affiliations:** 1 Istituto di Chimica dei Composti Organometallici (ICCOM), Consiglio Nazionale delle Ricerche (CNR), via Madonna Del Piano 10, I-50019 Firenze, Italy; giovanni.lapenna@cnr.it; 2 Section of Roma-Tor Vergata, Istituto Nazionale di Fisica Nucleare (INFN), via della Ricerca Scientifica 1, I-00133 Roma, Italy; 3 Istituto di Chimica dei Composti Organometallici (ICCOM), Consiglio Nazionale delle Ricerche (CNR), c/o Dipartimento di Chimica “Ugo Schiff”, via Della Lastruccia 13, I-50019 Firenze, Italy

**Keywords:** amides, carbanions, C-H acidity, nitro-aci tautomerism, molecular dynamics, density-functional theory

## Abstract

Many computational methods have been applied to interpret and predict changes in reactivity by slight modifications of a given molecular scaffold. We describe a novel and simple method based on approximate density-functional theory of valence electrons that can be applied within a large high-performance computational infrastructure to probe such changes using a statistical sample of molecular configurations, including the solvent. All the used computational tools are fully open-source. Following our previous application, we are able to explain the high acidity of C-H bond at α position in nitro compounds when the amide linkage an ammonium group is inserted into the α substituent.

## 1. Introduction

Acidity is an important characteristic of nitro compounds, and it determines, to a considerable extent, their reactivity [1,2]. A major contribution to the acidity is due to C-H bond geminal to the nitro group [3]. Generation of carbanions is a fundamental step in organic synthesis, and the nitro group is widely used to such purpose [4,5,6]. The heterolytic cleavage of activated C-H bonds has been investigated for a long time [7], in particular for nitro compounds, where the so-called “nitroalkane anomaly” was immediately recognized and related to solvent effects [8]. The behavior of Brønseted organic acids where the proton is released by C-H bonds has been interpreted in terms of electron attractive properties of substituents [9,10]. Nitro compounds escape from simple interpretations and, indeed, provide unusually strong acidity to C-H bonds in water. This effect is difficult to predict and interpret. Perturbative approaches based on Hammett–Taft equation have been used to predict quantitative structure–activity relationships. A closely related example was that of nitroalkanes [11]. Similar correlation approaches were used to predict the acidity constant values of organic compounds in organic and aqueous solutions [12,13,14]. Due to intrinsic limitations of such methods, and thanks to the advent of efficient computer hardware and software, researchers could explain extreme cases of particular interest by using, for instance, density-functional theory (DFT) [15]. Yet, the inclusion in such methods of explicit models of solvent is missing. Electronic effects due to groups in the solute and solvent molecules become linked together, and many atomic and electronic variables must be handled in a high-performance computational environment.

In our previous work [16], we addressed this issue by measuring apparent ionization constant ($pK_{a}$) for a series of substituted nitromethanes. We found that the amide moiety has a strong effect on the Cα-H acidity. Models including the water molecules interacting with the solute allowed to compare the contribution of intramolecular interactions to interactions with structured water layers. The models paved a way to answer the question about which are contributions more efficient to enhance the acidity of the geminal C-H bond: electronic effects of NO_2_ and R_2_NCO groups in to the solute; hydrogen bonds with water molecules involving the same groups and stabilizing different tautomers; and both effects eventually working together.

One particular finding provided by the previously applied models was that the hydronium species (H_3_O^+^) formed in the water solvent is different depending on the nitronate species that is formed as product. When the solute is more hydrophilic, the presence of an hydronium close to the solute decreases the potential energy of the product. On the other hand, when the solute is more hydrophobic, the hydronium species becomes far from the solute and it does not decrease the energy of the product. The observations indicate that the nature of the substituent, enhancing the acidity of Cα-H, should be hydrophilic in order to increase the probability of persistent hydronium species in the nearby of the solute. The presence of the amide moiety as substituent in the geminal position to the nitro group greatly enhances the Cα-H acidity, provided the amide substituent be hydrophilic. The model was able to predict the increase in Cα-H acidity of the secondary methyl amide CONHCH_3_ compared to tertiary CON(CH_3_)2, on the basis of the increase in hydrophilicity. The further modification of the amide side-chain was clearly indicated as the best solution to increase the C-H acidity.

**Figure 1:**
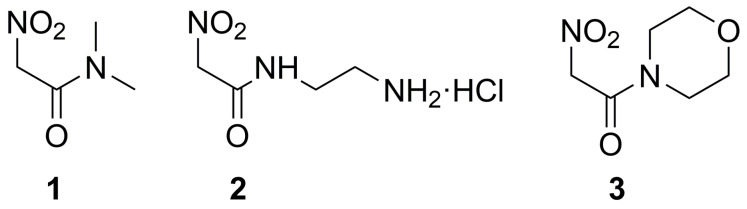
Nitroacetamides studied in this work: **1**: *N*-dimethyl-2-nitro-acetamide. **2**: *N*-(2-amino-ethyl)-2-nitro-acetamide hydrochloride. **3**: 1-morpholin-4-yl-2-nitro-ethanone.

Therefore, in this work, we have determined by potentiometric titrations the dissociation constant of compounds **2**–**3** in which the nitro group is combined with substituted amides of different hydrophilicity, as shown in Figure 1. The two new compounds are compared, as in the previous case, to the reference compound **1**.

If the nitro group confers high acidity to the carbon in α position, the amide moiety and its side chain will give the compound a high solubility in water and possibly an even higher acidity to the C(α)-H group.

The prototropic tautomerism of these compounds, already discussed in our previous work, is even more intricate for the new compounds investigated here. In secondary amides, like **2**–**3**, the labile amide proton is involved in *amide*/*iminol* tautomerism, which becomes additional to the *nitro*/*aci* tautomerism of previously investigated nitro compounds. Also, in **2**, the ammonium group can release the proton, contributing to apparent acidity.

The different effects, prototropic, electronic, steric, and those induced by the water solvent, are analyzed in detail, thanks to an approximated quantum mechanical model that we used to interpret the change in acidic constant along the series of studied compounds. We adopted the density-functional tight-binding (DFTB) approximation, which allows the simulation at experimental conditions (water density and temperature) of realistic trajectories where Hα proton is extracted by the Cα atom. The interatomic forces are calculated at a level of approximation almost equivalent to the generalized gradient approximation (GGA) to the exchange-correlation potential in density-functional theory, in this case the Perdew–Burke–Ernzerhof (PBE) approximation [17]. The adopted approximations to the Hamiltonian allow to simulate hundreds of processes of extraction for models of several thousands of atoms in periodic boundary conditions (typical of condensed matter), thus explicitly including a statistical analysis of the interactions with water molecules in the solvent. The latter are found critical to decrease the dissociation constant, up to values representing a vary low value for a C-H bond in water solution.

## 2. Results

### 2.1. Experimental Ionization Constant ($pK_{a}$)

In Table 1 and Table 2, we display the values obtained by titration of compounds **2** and **3**, providing by arithmetic mean $pK_{a}=$ 4.76 and 6.91, respectively. The $pK_{a}=$ 7.23 for compound **1** was determined in our previous work using the same procedure [16].

**Figure 2:**
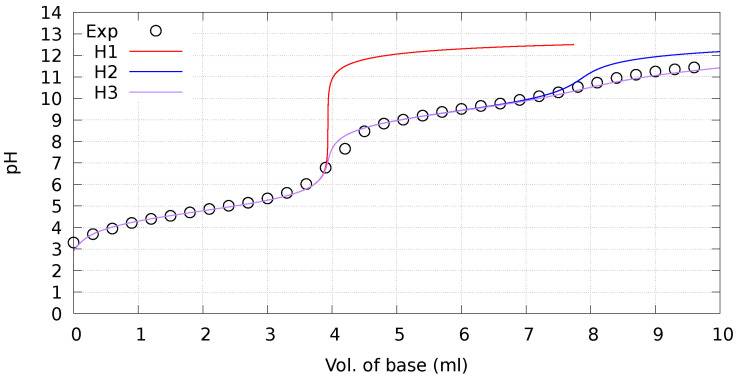
Titration curve of **2**, see Methods (Section 4) for concentrations. Exp.—This work. Theoretical curves: **H1**—monoprotic dissociation, $pK_{a}=$ 4.76; **H2**—biprotic dissociation, additional $pK_{a,2}=$ 9.4; **H3**—triprotic dissociation, additional $pK_{a,3}=$ 11.5.

The measured $pK_{a}$ for nitroacetamide **2** is a surprisingly small value, being comparable to acetic acid in water [18]. To confirm that the apparent $pK_{a}$ can be attributed to C-H bond dissociation, the full titration curve was analyzed as in the following. We modeled the titration curve of **2** as a single polyprotic species. In Figure 2, the data are compared to monoprotic (H1), biprotic (H2) and triprotic (H3) curves (see Methods (Section 4) for details). Biprotic curve is obtained with the second dissociation constant $pK_{a,2}=$ 9.4. This $pK_{a,2}$ value is consistent with values measured for lysine when bound into peptide segments where the side-chain is accessible by water [19,20]. The addition of a third dissociation constant $pK_{a,3}=$ 11.5 well reproduces the measured data, but the change with respect to the biprotic model is very small. Since the apparent dissociation constant of amides is almost non measurable in water solution ($pK_{a}$∼15), the latter dissociation can be tentatively attributed to an unusually high weight of the iminol tautomer of the amide group. In compound **2**, the iminol tautomer might be present with significant weight, even if in secondary amides it is estimated to be 10${}^{-8}$ times the amide [21]. However, such unusual propensity of amide proton dissociation was observed in water solution only in the presence of strong Lewis acids, like metal ions, that stabilize the negatively charged amide group. In the following, we assume the ammonium and amide groups of **2** protonated as in the nitro reactant when modeling the dissociation process of the Cα-H group. This assumption is required for the atomistic models described in the following.

### 2.2. Computed Ionization Constant

The external steering potential of Equation (2) (see Section 4) was able to produce the addressed reaction, that is the deprotonation of Cα, in a number of sampled trajectories. The classification of trajectories for the two studied compounds is reported in Table 3.

In both compounds, a number of trajectories diverge, because the constant time-step used in the Born–Oppenheimer molecular dynamics (BOMD) simulation is too long to keep the system in the electron ground state. These trajectories are classified as “faults” and are excluded from the statistics. We notice that the ratio between the number of reactive trajectories over the total number of successful trajectories is significantly larger for compounds **2** and **3** than **1**, 0.18 and 0.11 compared to 0.07, respectively. As explained in “Materials and Methods” (Section 4), we call this ratio the fraction of achieved products (or reactive ratio) obtained in the steering experiment, $x_{p}$ (see Equation (10)). The larger value of $x_{p}$ for **2** compared to **1** is a clear indication that compound **2** has a larger propensity to react than **1**. Even if a rigorous analysis should be performed, this parameter is related to α parameter of ref. [7].

**Figure 3:**
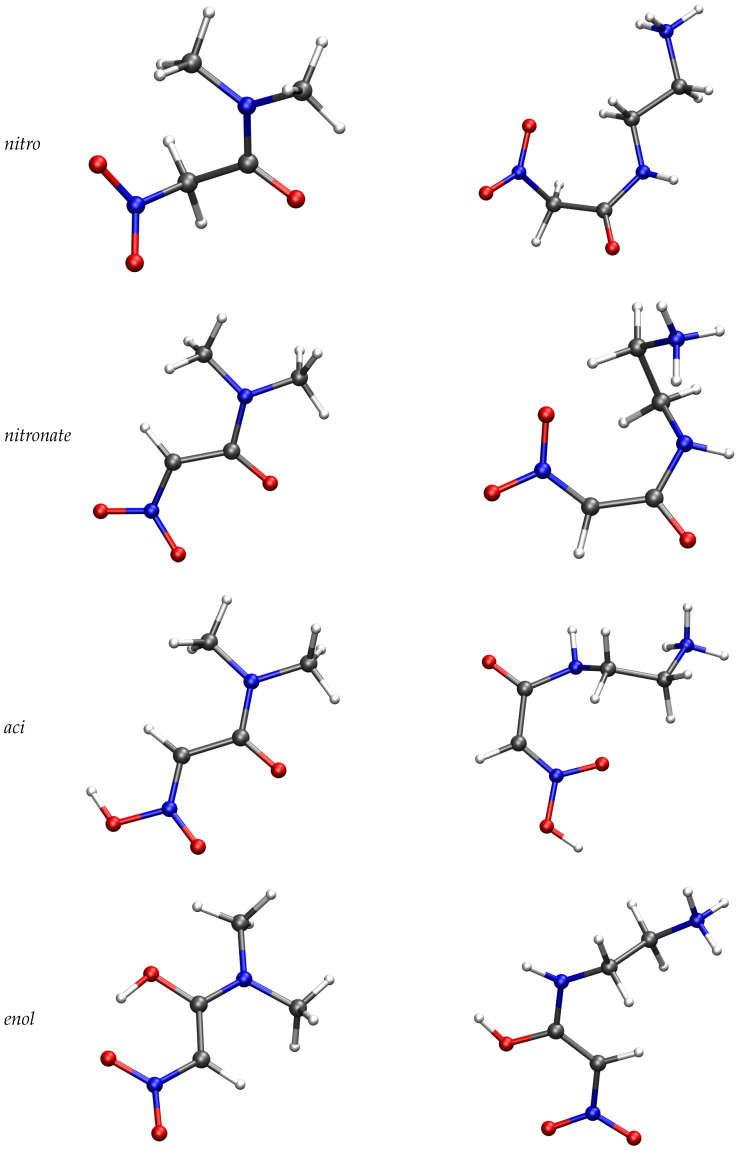
Model structures of compounds **1** (**left**) and **2** (**right**). Configurations are local energy minima of solute in implicit solvent (see Methods (Section 4)) obtained in the density-functional tight-binding approximation. Energies relative to reactant are in Table 4.

In Figure 3, selected configurations of the solute molecule are displayed for **1** (middle) and **2** (right).

The extracted proton is in the solvent bath (not displayed), and the detailed characterization of the final state (the product state) will be described in a following subsection.

**Figure 4:**
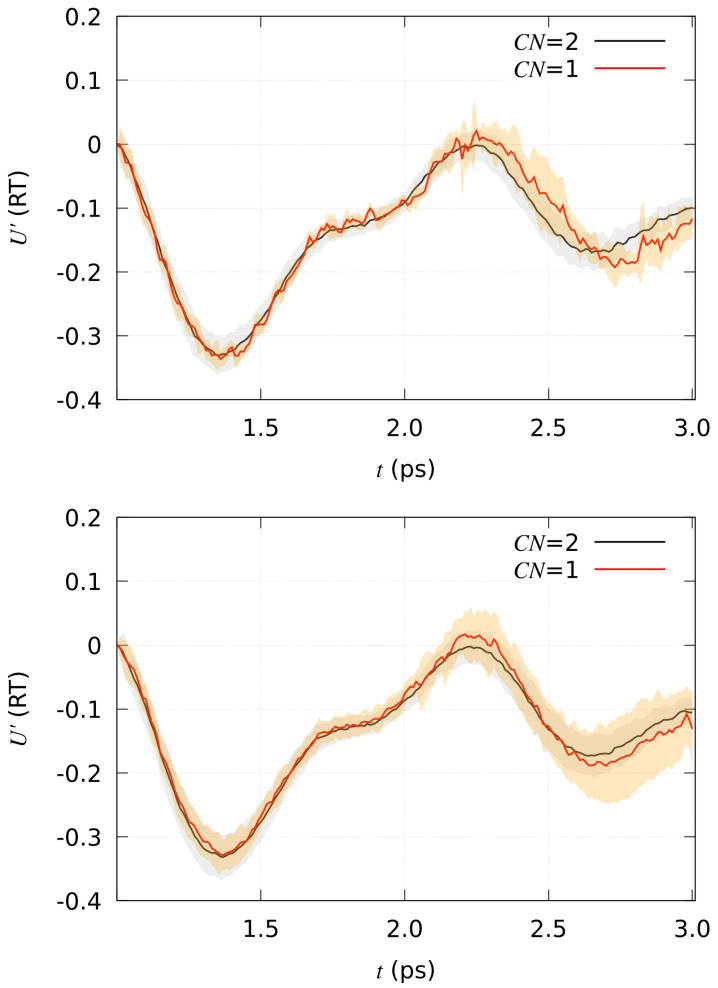
Time evolution of $U^{\prime }$ in Equation (9). Model **1** (**top**); model **2** (**bottom**).

As for the analysis of the energy change upon chemical reactions, the time evolution of $U^{\prime }$ (see Section 4) is plotted, once averages are performed within each class of trajectories, in Figure 4.

The shadowed band is displayed using the root-mean square deviation obtained in the same averaging process. The black and red curves (with gray and orange shadowed bands) are, respectively, $U_{r}^{\prime }$ and $U_{p}^{\prime }$ of Equation (10).

Some features can be observed in Figure 4. An increase in $U_{p}^{\prime }$ with respect to $U_{r}^{\prime }$ is visible for both compounds when the reaction occurs; that is, for most of the reactive trajectories at t= 2.2 ps from the beginning of steering process (time 1.0 in the figure, that is after 1 ps of thermal equilibration). The main difference between compound **1** and **2** is that in compound **2** the difference in energy, $U_{p}^{\prime }\left(t\right)-U_{r}^{\prime }\left(t\right)$, becomes negligible in a short time, with reactive and non reactive trajectories mostly overlapped after a short transition time of about 0.2 ps during the steering process. Compound **1** displays no way to dissipate the energy acquired by the steering process, while **2** rapidly finds configurations where water molecules hook the expelled proton and, at the same time, the solute molecule in the product state accommodates, on average, the stress induced by the proton release. The energy of the final product state, $U_{p}^{\prime }$, is smaller than that of the average of non reactive configurations, $U_{r}^{\prime }$, as it is shown by the final evolution of $U^{\prime }$ displayed in Figure 4. The average in Equation (10) is performed using the last 200 points of the trajectories.

The application of Equation (10) provides the data reported in Table 5 for compounds **2** and **3**, using as reference compound **1**.

The values compare fairly well to the values measured by titration. What is particularly important is the sign of the change, which is well captured by the simulations. The major limitation of the procedure preventing to obtain a more precise measure of the energy difference between product and reactant is the actual temperature of the system, as this parameter is produced by molecular dynamics (MD) simulation. The temperature displays, for such small samples of ∼1000 atoms, a slow oscillation of about 200 K, due to the deterministic thermal bath required by BOMD simulations at constant average temperature. The actual temperature must not be confused with the temperature of the bath, that is imposed by the thermostat and it is the average value of the actual temperature. However, the time evolution of the potential energy of the system is affected by the oscillation of the actual temperature, while the average potential energy is a consequence of the constraint for the average thermodynamic parameters imposed to the equation of motion. Such large and slow temperature fluctuation requires very long simulation time to obtain fully equilibrated samples in the final state. The computational wall-clock time to obtain a single trajectory of 3 ps is for a system of 1268 atoms about 7 h. The thermal equilibration requires a ns time-scale, meaning, in terms of computational wall-time, 1000 times the above wall-time required by ps time-scales. Conversely, the usage of 100 trajectories collected in a parallel computational architecture allows to circumvent this limitation without resorting to huge computational times, provided an empirically equilibrated sample of reactant is used to start with.

Therefore, by comparing reactive to non reactive trajectories, the statistical limitation is partially overtaken, because we are comparing energies measured at very similar temperature. This technique, therefore, requires a particular care in keeping in phase the time evolution of atomic velocities.

### 2.3. Trajectories Analysis

In the following, we analyze in more details the distance between the hydrated proton in the solvent and the solute atoms when the reaction occurs. This analysis is also performed for the few successful trajectories where the proton is forced to move from the water bath back to the solute. The latter trajectories represent possible atomic pathways of the backward reaction.

**Figure 5:**
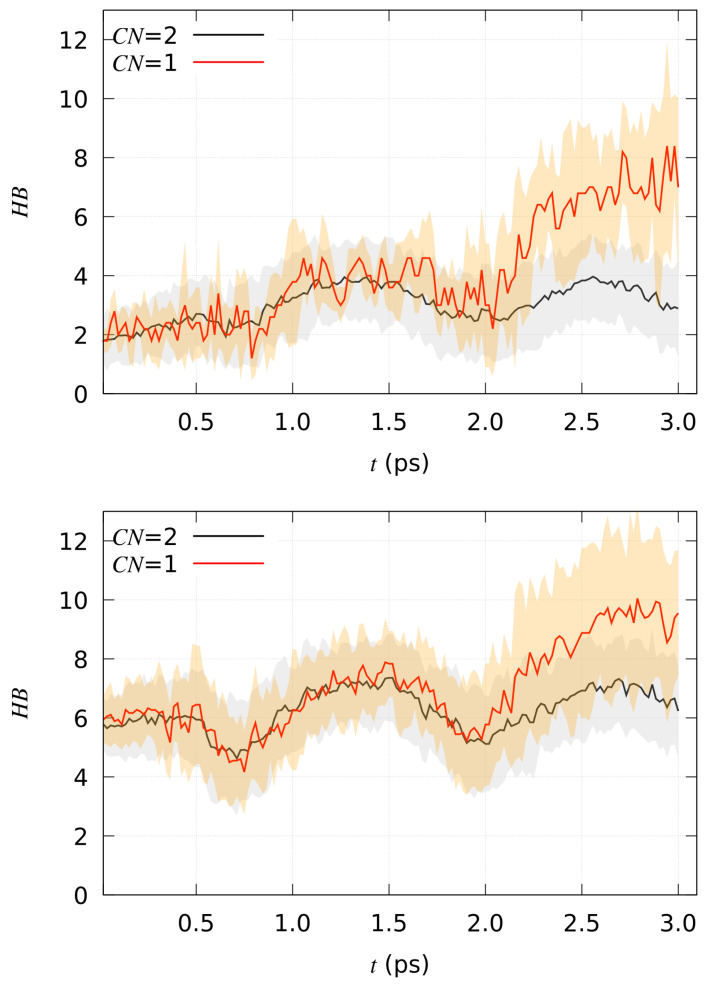

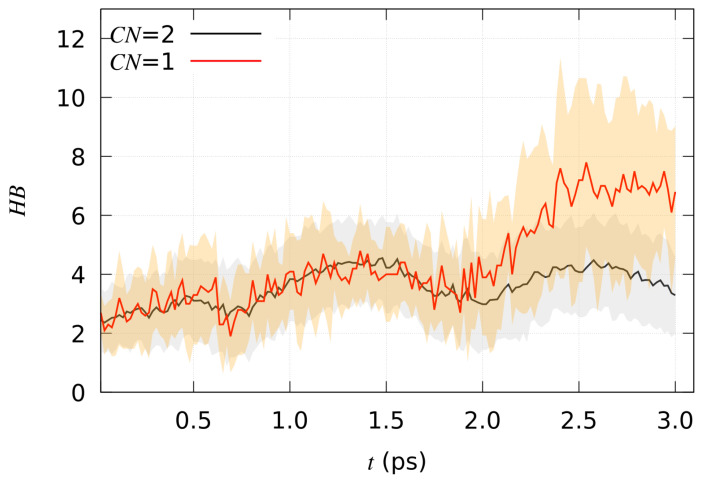
Time evolution of the number of hydrogen bonds (HB). Model **1** (**top**); model **2** (**middle**); model **3** (**bottom**).

In Figure 5, the time evolution of the number of hydrogen bonds between the solute and the water solvent are displayed as a function of time for all the simulated models **1**–**3** in the forward process. Reactive and non-reactive trajectories are analyzed separately and displayed as red and black curves, respectively, as in Figure 4. In the figure, the time-evolution during the first ps of thermal equilibration (no steering potential) is also displayed. It can be noticed that in compound **2**, the solute is more hydrophilic when in the reactant state, where a larger number of hydrogen bonds are sampled since the beginning compared to **1**. The number of hydrogen bonds in the final product is similar in the two compounds, while in reactants, the difference is larger: about four less hydrogen bonds in **1** than in **2**. We argue that in compound **2** the reactant is “prepared” for the reaction, a property that is confirmed by the larger number of reactive pathways in **2** than in **1** (see Table 3). Again, there is an effect of average velocities (instantaneous temperature) displayed as an oscillation in the average number of hydrogen bonds. This effect is associated with the oscillatory change in structure in the solvent bath. However, the change in the number of hydrogen bonds due to proton extraction from position α is visible, occurring after 1 ps of application of the external force (time 2 ps).

The behavior of compound **3** is intermediate between the two compounds **1** and **2**. As for the change in energy $U^{\prime }$, affecting the change of $pK_{a}$ reported in Table 5, we notice a slightly smaller shift of $pK_{a}$ towards low values. On the other hand, as for the value of $x_{p}$ (Table 3) and the number of hydrogen bonds in the reactant state, compound **2** is similar to **1**, because of the bulky side-chain of the amide group.

This intermediate situation of compound **3** within **1** and **2** is also displayed by other structural properties analyzed in the reactant state.

**Figure 6:**
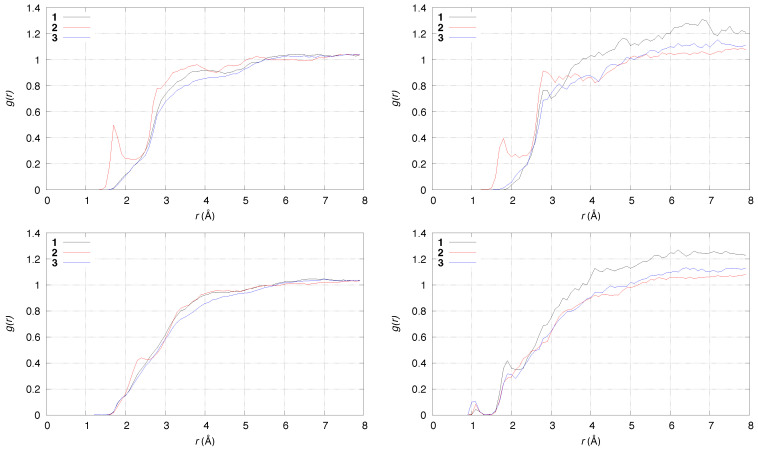
Radial distribution function of solute-O(water) (**top**) and solute-H(water) pairs (**bottom**). (**Left**)—reactant state (0–0.5 ps); (**right**)—product state (3.5–4.0 ps). **1** is black; **2** red; **3** blue.

In Figure 6, the radial distribution function for atomic pairs involving solute atoms and, respectively, O (top panels) and H (bottom) atoms of water molecules are displayed. The leftmost peak in the reactant state (left panels) displayed by **2** is due to the hydrogen bond between the N of the ammonium group (donor) and water molecules (acceptor). This hydrogen bond, not displayed by compounds **1** and **3**, is only slightly weaker in the product state (right panels). The leftmost peak in the product state for X-H(water) pairs (bottom-right panel, X any of the solute atoms) is due to the few configurations where the Cα-Hα bond is not yet broken. These configurations are sampled because of the 0.5 ps time-window chosen to represent product state. The **3** compound, like **1**, does not sample configurations with strong hydrogen bonds (see also Figure 5). An interesting feature of the radial distribution function in the product state is the deviation from the ideal behavior at long distances (8 Å), where the g(r) function should approach the value of one. This behavior is due to the destabilization of bulk water when one proton is moved from the solute to the solvent. The settling of the cell size in the simulation of the product to accommodate the change of pressure should be performed, but the small cell size used in our models in the DFTB approximation does not allow such task because actual pressure oscillation is very large. Also, an empirical approximation of water with an excess proton is not feasible with the available force-fields. Even if this limitation of the model of interatomic forces is clearly shown, we must notice that this event is more significant for compound **1**, thus showing that the water layer is more destabilized when **1** is deprotonated than in the other two cases.

Further information about the events occurring upon nitroacetamide deprotonation at α position can be obtained comparing the backward process performed for **1** and **3** compounds. When the backward transformation is attempted on **1**, among the five products obtained by the forward process, only one trajectory succeeds in transferring the proton back to the solute in the *aci* form, that is the proton becomes bound to one of the nitro O atom. The successful backward trajectory displays transient formation of the *enol* form with a planar ring involving the nitro group, as shown in Figure 3 (middle/bottom panel). The other four trajectories are forced by the external bias to remove one O-H bond in water, but with no success of proton addition to the solute in 2.5 ps.

The compound **2** behaves similarly. Among the 18 configurations obtained in the product state, two fail in keeping the ground state, thirteen remain in the product state despite the external bias applied for 2.5 ps, and three succeed in transferring the proton to the solute. In two cases, the *aci* form is obtained, while in one case the *enol* form is obtained, with the proton binding the carbonyl O atom of the solute. These trajectories never form configurations with the added proton forming a planar 6-members ring like for compound **2**. Despite the fact that these configurations can transiently stabilize the proton transfer from the α position towards the solvent (and the reverse), they are very rarely observed in simulations because of the thermal fluctuations affecting the solute. We argue that *aci* and *enol* forms of the reactant do not statistically contribute to Cα acidity.

Other intramolecular interactions can contribute to the stabilization of the nitronate product. The most evident interaction is the electrostatic attraction in **2** between the positively charged ammonium group and the negatively charged nitronate group. This interaction causes the compaction of the molecule. In order to monitor this event, we measured the time-evolution of the N-N distance (*d*, hereafter) along with the 98 collected trajectories of **2**. Among the 80 non-reactive trajectories, 22 display the distance smaller than 4.5 Å at several configurations. On the other hand, in 11 of the 18 reactive trajectories the distance reaches values smaller than 4.5 Å, and in nine of these, the molecular compaction occurs when the system is in the nitronate form. These data represent a low chance to compact the molecule when in the reactant nitro form and a moderate propensity to compact when in the nitronate product. Averaging over the trajectories at given times this effect is, however, small. After 1 ps of thermal equilibration of reactant, we observe: 〈d〉 = 5.8 ± 0.9 Å for non-reactive trajectories; 〈d〉 = 5.7 ± 0.8 Å for reactive. At the end of the application of the external bias (3 ps): 〈d〉 = 5.8 ± 0.7 Å for non-reactive; 〈d〉 = 5.5 ± 0.6 Å for reactive. The small change in the average means that thermal fluctuations at room conditions in water solution almost cancel the effects of electrostatic attraction between the partial charges distributed over the molecule, even in the nitronate product state.

The potential energy of different chemical species contributing to the acid-base equilibrium is analyzed in the following. In Table 4, we display the changes in electron energy (that is the potential energy of the BOMD simulation) moving from the initial reactant state to several final states. Final states are characterized by the chemical bonds as they are identified in each configuration. These are the species displayed (for compounds **1** and **3**) in Figure 3: the *nitro* form, that is the reactant as it was built by the empirical initial simulations (this is the zero reference of energy change); the *aci* form, when one proton is released by the Cα and one proton is finally bound to the O in the nitro group; the *enol* form, when one proton is released by the Cα and one proton is finally bound to the O in the carbonyl group; the *nitronate* form of the product, when one proton is released by the Cα and it becomes bound to water molecules in the solute environment. The energy is computed extracting one configuration among those sampled in each state and finding the local energy minimum once all the solvent molecules and the periodic boundary conditions are removed. The latter conditions are replaced with the solvation energy computed by the generalized-Born solvent-accessible surface area (GBSA) method [22], as it is implemented in the DFTB+ code (see Section 4).

The numbers are obviously affected by large errors due to the bias of extracting configurations within a sampling at room-conditions in the ps time-scale. Interestingly, the stabilization of the *aci* form in **2** indicates a large contribution to acidity of this form, which is in the same direction of experiments. However, the negative energy change to obtain the nitronate product and the small energy of *enol* forms show that in the implicit solvent model many contributions are missing.

The above data indicate that the explanation of the change in acidity of Cα that is observed in experiments is in the different probability of the configurations, rather than in the relative energy of necessarily selected configurations. In this respect, the attempt to compute the change in the probability of success of the extraction process seems to contain information that is missing in the energy alone.

## 3. Discussion

The search for a chemical descriptor of nitroacetamide compounds able to predict the anomalous acidity of the C-H bond in α position to the nitro group has been active for a long time. In this work, we continued the detailed analysis of solvent effects in the process of Hα extraction that we first introduced in ref. [16]. In particular, we used a simplified model of atomic interactions, approximated at the density-functional tight-binding level, but extended to the analysis of 100 extraction trajectories.

Even if a rigorous statistical analysis of the transition state is not possible, because of the large number of configurations required, the role of water molecules in stabilizing intermediate configurations between reactant and product state has been confirmed. The usage of 100 configurations allows to define the parameter $x_{p}$, that we called the reactive ratio, as a valid chemical descriptor. The latter parameter combines the structure of the solute, including electronic effects of substituents, and the chance for water molecules in the first solvation layer to efficiently hook the proton extracted from the Cα atom.

The reactive ratio is strongly related to the average number of hydrogen bonds in the reactant state, but it contains more information that is able to discriminate the morpholinyl from the amino-ethyl side-chain. The relative order of $x_{p}$ values (last column of Table 3) is a good representation of the relative change in $pK_{a}$ found in experiments.

Straight-forward prediction tools available in the literature or commercially for such changes in $pK_{a}$ seem not be able to capture the importance of changes in the interactions between the solute and solvent molecules. For example, the prediction of ACDLabs™ (Toronto, ON, Canada) (available in SciFinder™, Columbus, OH, USA) for $pK_{a}$ are 5.99, 5.72, and 6.94 for **1**, **2**, and **3**, respectively. The prediction is particularly wrong for **1**, probably due to the low prediction of the water layer destabilization upon proton extraction, which is in the product state. The octanol/water partition, logP = $\log(\frac{{\left[X\right]}_{\mathrm{octanol}}}{{\left[X\right]}_{\mathrm{water}}})$ is, at T= 25 °C for **1**, −0.538, which is even lower than that for **3** (−0.384). This parameter is used to rank reactant molecules in terms of stabilization due to favorable interactions with water. However, as expected for the series of “anomalous” nitro acetamides, these interactions deserve a detailed description that is missing in the characterization of reactant state only.

## 4. Materials and Methods

### 4.1. Preparation of Nitroacetamides and Their Characterization

Melting points were determined in capillaries with a Büchi 510 apparatus and are uncorrected. Chromatographic separations were performed on silica gel 60 (40–6.3 mm) with analytical grade solvents, driven by a positive pressure of air; R${}_{f}$ values refer to TLC (visualized with UV light and/or by dipping the plates into a solution of permanganate followed by heating with a heat gun) carried out on alumina-backed plates coated with 25 mm silica gel (Merck F254), with the same eluant indicated for the column chromatography. For gradient column chromatography R${}_{f}$ values, refer to the more polar eluant. Solvent removal was performed by evaporation on a rotavap at room temperature. ${}^{1}$H- and ${}^{13}$C-NMR spectra were recorded with a Varian Mercuryplus 400 spectrometer. The ${}^{1}$H-NMR data are reported as multiplicity (s = singlet, d = doublet, t = triplet, m = multiplet or unresolved, br = broad signal), coupling constant(s) in Hz, integration. Multiplicity of the ${}^{13}$C-NMR signals and assignments were determined by means of gHSQC and gHMBC experiments. Chemical shifts were determined relative to the residual solvent peak (CHCl_3_: δ = 7.24 ppm for ${}^{1}$H-NMR and δ = 77.0 ppm for ${}^{13}$C-NMR). ESI (electron spray ionization) mass spectra were obtained with a ThermoFisher LCQ-Fleet ion trap instrument and spectra were registered with ESI${}^{+}$ techniques. IR spectra were recorded with a Perkin–Elmer 881 spectro-photometer; bands are characterized as broad (br), strong (s), medium (m), or weak (w). Elemental analyses were obtained with an Elemental Analyser Perkin–Elmer 240C apparatus. The pH values were determined with a CyberScan510 pH meter produced by Eutech instruments.

Nitroacetamides **1**–**3** were prepared by aminolysis of methyl nitroacetate [23].

#### 4.1.1. *N,N*-dimethyl-2-nitroacetamide (**1**)

NHMe_2_ (40% solution in H_2_O, 10 mL) was added to cooled (ice bath) methyl nitroacetate (0.5 g, 4.20 mmol) and the mixture maintained on stirring at room temperature. After 6 days, the excess of amine was removed by a stream of nitrogen. Then, a solution of HCl (20% in H_2_O) was dropwise added to pH = 2. The mixture was extracted with Et_2_O (4 × 35 mL) and the combined organic layers were dried on Na_2_SO_4_, filtered, concentrated under reduced pressure and the solid residue purified by flash chromatography on silica gel (CH_2_Cl_2_/Methanol 100:1, then CH_2_Cl_2_/CH_3_OH 80:1, R${}_{f}$ = 0.17). Product **1** was white powder (0.424 g, 69% yield). Melting point (M.p.) 78–79 °C (Lit. [24] 78 °C). NMR spectra are displayed in Figure S1 (see Supplementary Materials). ${}^{1}$H-NMR (400 MHz, CDCl_3_): δ = 2.99 (s, 3 H, C*H*3), 3.02 (s, 3 H, C*H*3), 5.27 ppm (s, 2 H, C*H*2NO_2_). ${}^{13}$C-NMR (100.59 MHz, CDCl_3_): δ = 35.9 (q, N*C*H_3_), 37.0 (q, N*C*H_3_), 160.6 ppm (s, *C*O); *C*H_2_NO_2_ carbon was not detected. IR (CDCl_3_): $\tilde{\nu }$ = 2934 (w), 1674 (s) [C=O], 1568 (s) [asym. O−N−O], 1408 (m), 1373 (m) [sym. O−N−O], 1262 (w), 1203 (w), 1149 cm${}^{-1}$ (w). MS (ESI${}^{+}$, MeOH): m/z (%) = 155 (100) [*M* + Na]${}^{+}$. Elemental analysis calcd (%) for C_4_H_8_N_2_O_3_ (132.12): C 36.36, H 6.10, N 21.20; found: C 36.38, H 6.10, N 21.17.

#### 4.1.2. * N*-(2-aminoethyl)-2-nitroacetamide hydrochloride (**2**)

This was prepared by aminolysis of methyl nitroacetate with ethylendiamine followed by a treatment with a 1 M methanolic solution of HCl, as previously reported [25]. White powder, m.p. 118–120 °C (dec.) (Lit. [25] 118–121 °C (dec.)). Elemental analysis calcd (%) for C_4_H_9_N_3_O_3_·HCl (183.59): C 26.17, H 5.49, N 22.89; found: C 26.46, H 5.93, N 22.45.

#### 4.1.3. 1-morpholin-4-yl-2-nitro-ethanone (**3**)

This was prepared by slightly modifying a previously reported procedure [26]. A mixture of methyl nitroacetate (0.5 g, 4.20 mmol) and morpholine (3.6 mL, 10 equiv.) was heated whilst stirring at 100 °C. After 2 h, the mixture was cooled and the excess of morpholine was removed under reduced pressure. Then, the solid residue was dissolved in 8 mL of water and a 20% HCl solution was added to pH = 2, cooling at ∼0 °C. The obtained white precipitate was filtered, washed with water until pH = 5 and dried to give **3** (0.256 g, 35%) as a white powder. M.p. 106–108 °C (Lit. [26] 107–109 °C). NMR spectra are displayed in Figure S2 (see Supplementary Materials). ${}^{1}$H-NMR (400 MHz, CDCl_3_): δ = 3.34–3.40 (m, 2 H, C*H*2N), 3.62–3.68 (m, 2 H, C*H*2N), 3.68–3.72 (m, 4 H, C*H*2O), 5.27 ppm (s, 2 H, C*H*2NO_2_). ${}^{13}$C-NMR (100.59 MHz, CDCl_3_): δ = 42.7 (t, *C*H_2_N), 46.1 (t, *C*H_2_N), 66.1 (t, *C*H_2_O), 66.4 (t, *C*H_2_O), 76.6 (t, *C*H_2_NO_2_), 159.3 ppm (s, *C*O). IR (CDCl_3_): $\tilde{\nu }$ = 2975 (w), 2927 (w), 2864 (w), 2864 (w), 1667 (s) [C=O], 1567 (s) [asym. O−N−O], 1447 (m), 1411 (w), 1373 (m), 1362 (m), 1315 (w), 1362 (m), 1315 (w), 1302 (m), 1236 (m), 1202 (w), 1116 (s), 1068 (w), 1039 cm${}^{-1}$ (w). MS (ESI${}^{+}$, MeOH): m/z (%) = 200 (100) [*M* + Na]${}^{+}$. Elemental analysis calcd (%) for C_6_H_10_N_2_O_4_ (174.15): C 41.38, H 5.79, N 16.09; found: C 41.23, H 5.75, N 16.26.

### 4.2. Determination of Ionization Constants (Apparent $pK_{a}$)

Ionization constants of nitro compounds **2** and **3** were determined in water by potentiometric titration using a glass electrode (method of partial neutralization).

The values of $pK_{a}$ were calculated according to the formula:

(1) $$pK_{a}=-\log\left(K_{a}\right)=\mathrm{pH}+\log\frac{\left[\mathrm{HA}\right]}{\left[A\right]},$$

where [HA] is the concentration of non-dissociated nitroacetamide, [A] is the concentration of its salt, and log is the base-10 logarithm. The usage of NaOH as strong base in titration, the low concentration, and the chlorohydrate salt used for **2** guarantee the complete dissociation in water at room conditions of all species investigated in this study. No precipitation was observed in any sample.

As for compound **2**, the estimate of the dissociation constants ($pK_{a,2}$ and $pK_{a,3}$ of the nitronate form was made by manually changing model titration curves once the dissociation constant of the nitro compound was measured as described above. To generate titration curves, we used the libraries developed for the R language [27] that implement the multiple chemical equilibria as described in ref. [28].

### 4.3. Calculation of Ionization Constants

The final goal of our models is to investigate the mechanism of the nitroacetamide deprotonation reaction within a density-functional theory (DFT) approximation of electrons in a system composed of the solute nitroacetamide compound and a sample of solvent water molecules. In our previous work [16], we applied an implementations of DFT suited for systems of several hundreds of atoms in order to obtain the energy change in the sample upon the transfer of one of the Hα protons into water. In this work, we opted for extending the usage of the semi-empirical model we used before applying DFT time-consuming calculations. In particular, we decided to extend the statistics required to compute the free energy change. Therefore, we performed 100 molecular dynamics (MD) simulations in the Born–Oppenheimer (BO) approximation and at room conditions (BOMD, hereafter) within a semi-empirical Hamiltonian describing atomic cores and valence electrons. The Hamiltonian of the system was based on the self-consistent charge density-functional tight-binding approximation [29] (DFTB), because geometrical parameters (like distances and angles) of minimal energy conformations are consistent with accurate DFT calculations for a large set of organic molecules, both isolated and in condensed phases. We used the DFTB+ code [30] for these simulations. The valence electrons of each atom are represented as *s* and *p* orbitals. In the following, we describe the details of all calculations.

#### 4.3.1. System Preparation

We built the initial solute nitro compound **2** according to standard geometrical parameters. Before merging the solute in the water bath, a number of solute configurations was built because, differently from our previous application, we prepared a larger sample of water molecules using 100 different initial configurations for the solute. We performed a self-avoiding random walk of the solute by randomly rotating all dihedral angles in the molecule. A Monte Carlo Metropolis test was performed using a random temperature in the range 10${}^{-3}$–10${}^{4}$ K, to exclude from the trajectory configurations with large repulsive energy.

As usual, to minimize finite volume effects, periodic boundary conditions in the tree directions of space were imposed to the system when interatomic forces were computed. To build the initial periodic simulation cells we proceeded as in the following.

We merged 100 of the resulting solute conformations into a snapshot of the sample of water molecules simulated by MD with the TIP3P interaction potential [31]. This step was made with the solvate program available in VMD [32]. The initial water sample is a cubic unit cell with the side of 6.571 nm containing 9261 water molecules, in a configuration extracted by the MD simulated trajectory in the NPT statistical ensemble with T= 300 K, P= 1 bar, and average density of 0.976 g/cm${}^{3}$. From this cubic sample, the program extracts all water molecules contained within 1.5 nm from the solute, excluding the water molecules with the O atom closer than 1.2 Å from any solute atom. The size of all cells (100 in number, one for each initial solute configuration) were about 30 Å. The number of water molecules around the solute and the size of the resulting orthorhombic cell change according to the initial solute configuration. A central point in our method is that all representations of the system is composed of the same number of water molecules. To accomplish the task of leveling the number of water molecules among the 100 built configurations, we iterated over applications of the solvate program, changing the size of the final system: when the number of water molecules was larger than the average value of 100 configurations, we decreased the size of the final cell; the opposite when the number was smaller. After 3–4 applications of this procedure, the final leveling was performed randomly deleting the few water molecules in excess or defect with respect to the average. The final number of water molecules in all samples was 416.

#### 4.3.2. Thermal Equilibration

Each of the samples that were prepared was equilibrated to achieve a configuration consistent with the thermodynamic state of the pH measurements. This task was performed with a mechanical force-field and classical molecular dynamics, before performing quantum-mechanical simulations. We used the code LAMMPS [33] and the OPLS force-field [34,35], with some additional parameters for the compounds of our study found in the literature [36].

The energy of the system was minimized via the conjugate gradient algorithm for 10,000 steps, in order to reduce the force initially acting on the atoms. Then, the MD simulation in the NVE statistical ensemble was performed for 10,000 steps, starting with velocities extracted from a Gaussian distribution at T= 50 K and with a time-step of 0.25 fs. During this stage, the temperature never reached values larger than 50 K, indicating the absence of close contacts between atoms. The velocity-verlet algorithm was used to integrate the equations of motion [37]. A second NVE stage of 20 ps was performed using geometrical constraints over all bond lengths involving hydrogen atoms [38], allowing a time-step of 2 fs. The MD simulation in the NVT statistical ensemble was then performed continuing the trajectory by using the Nosé–Hoover thermostat [39] at T= 150 K for 200 ps, followed by 200 ps at T= 300 K. A unique effective mass corresponding to a coupling constant of 10 THz was used for the thermostat. Finally, a 1-ns MD simulation in the NPT statistical ensemble [40] was performed to settle the water density around the solute, a critical parameter for all the following quantum-mechanical calculations. The isotropic scaling of simulation cell was used together with a damping time of 1 ps. The last collected configuration was used as initial configuration for the following quantum-mechanical calculations, keeping the cell size constant as in the final point.

#### 4.3.3. Density-Functional Tight-Binding MD

The DFTB+ code (version 2022 [41]) was used for all of the following calculations in the density-functional tight-binding approximation. The energy of the system was minimized via the LBFGS algorithm [42] for 100 steps, in order to reduce the force initially acting on the atoms when passing from the empirical model to the quantum-mechanical one. Then, the MD simulation in the NVT statistical ensemble was started using the atomic velocities as extracted from the final configuration of the previous empirical step. By this procedure, a long thermal equilibration in the quantum-mechanical model was partially circumvented. Finally, the velocity-verlet algorithm was used to integrate the equations of motion, together with the Nosé–Hoover thermostat, as in the previous empirical simulation stage. The time-step for the Born–Oppenheimer MD simulation was 1 fs. Simulations in the NPT statistical ensemble are extremely slow even in the DFTB approximation. Therefore, we decided to keep the volume constant as it was found in the empirical model of reactant.

### 4.4. Pulling αH in DFTB Models

We performed pulling experiments in order to explore possible pathways for the mechanism of Cα-H bond breaking, together with the formation of O-H bonds in the water layer around the solute. The procedure was identical to that performed in the DFT model in our previous application [16]. We used the Plumed code (2.6 version [43]), as it has been implemented as an external force to DFTB+.

We defined a collective variable as the Cα coordination number CN according to Equation below [44]:

(2) $$\begin{array}{ccc} CN & = & \sum_{i,j}s_{i,j}\\ s_{i,j} & = & 1\;\;\;\mathrm{if}\;r_{i,j}\leq 0\\ s_{i,j} & = & \frac{1-{\left(\frac{r_{i,j}}{\sigma }\right)}^{6}}{1-{\left(\frac{r_{i,j}}{\sigma }\right)}^{12}}\;\;\;\mathrm{if}\;r_{i,j}>0\\ r_{i,j} & = & |r_{i}-r_{j}|-d_{0}\;, \end{array}$$

where the index *j* runs over the α H (two) atoms of the solute and *i* indicates Cα. The actual value of CN can be therefore manipulated by defining an external force as the derivative of an external harmonic potential $U_{e}=\frac{k}{2}{\left(CN-CN_{0}\left(t\right)\right)}^{2}$. By progressively decreasing $CN_{0}$ with the simulation time *t*, we allow the smooth release (when the target $CN_{0}$ value becomes lower than the actual value of CN) of one of the two α H atoms. With this procedure, and due to the presence of the explicit water molecules in the model, the C-H bond is broken and, when available, a new O-H bond is formed in the water layer around the solute. The parameters $d_{0}$ and σ in Equation (2) were set to 1.1 Å and 0.5 Å, respectively. The parameter *k* was set to 1250 kcal/mol. The value of $CN_{0}$ is moved from 2 to 1 at the rate of 1 CN value in 2 ps. The pulling of α H was performed in 2 ps after an equilibration stage of 1 ps.

A second pulling experiment was performed to bring back the proton from the water reservoir to any of the solute atoms. This stage is indicated as backward process in the solute deprotonation reaction (the forward process). The backward process is a tentative process of reconstructing the solute as in its original reactant state. As for this backward pulling experiment, the index *i* in Equation (2) runs over the O atoms of the water molecules, while *j* runs over all the H atoms potentially bound to water. The H atoms, therefore, include the Hα atoms of the solute. The $d_{0}$ parameter was 0.98 Å, the latter the equilibrium distance for O-H in water measured as the average over DFTB simulations of water. The $CN_{0}$ parameter was decreased from the initial value (2 × 416 + 1) to that of bulk water (2 × 416), at the same speed of the forward process. The force constant of the external bias (*k*) was set to a smaller value, 1/100 of the forward process, to avoid strong increase in temperature of the system. These events can occur in the backward process because of the many chances to remove a O-H bond in water molecules, the latter being more than 100 times in number than the solute molecules.

### 4.5. Data Analysis

The aim of our study is at comparing measured changes in $pK_{a}$ between different species to results obtained by statistical analysis of computational models. The following equations are applied:

(3) $$\begin{array}{ccc} \Delta pK_{a} & = & -\log K_{a}\left(2\right)+\log K_{a}\left(1\right) \end{array}$$

(4) $$\begin{array}{ccc} & \;\;\;\;\;= & \frac{1}{\ln(10)}\left[-\ln K_{a}\left(2\right)+\ln K_{a}\left(1\right)\right] \end{array}$$

(5) $$\begin{array}{ccc} & \;\;\;\;\;\;= & \frac{1}{\ln(10)}\left[\Delta G\left(2\right)/RT-\Delta G\left(1\right)/RT\right] \end{array}$$

(6) $$\begin{array}{ccc} & \;\;\;\;∼ & \frac{1}{\ln(10)}\left[\Delta U\left(2\right)-\Delta U\left(1\right)\right]/RT \end{array}$$

(7) $$\begin{array}{ccc} & \;= & \frac{1}{\ln(10)}\left(\Delta \Delta U\right)/RT \end{array}$$

where *G* is the free energy at standard conditions. We shall adopt the assumption that the change in free energy divided for RT is the change in the potential energy $U^{\prime }=U/RT$.

The potential energy *U* during the BOMD simulation performed within a (though approximated) DFT method, is the total electron energy computed at each point of the trajectory in time. The value of *U* for the entire system is a function of many degrees of freedom when atomic samples are in the liquid state at room conditions. The large variation in *U*, even in a non reactive trajectory, is due to many sources:

1. The simulation of the stochastic regime performed with a deterministic trajectory;
2. The high frequency motions sampled in the ps time-scale of BOMD simulation.

In a reactive trajectory, the change in *U* due to the reaction (the reactive event) is hidden within the equilibrium fluctuations summarized above.

In the following we describe the method we used to extract the change in *U* due to the reaction. We shift the potential energy *U* for a quantity approximately representing the contributions of the vibrational degrees of freedom in the system. This contribution is:

(8) $$U_{vib}=N_{deg}RT/2\;\;,$$

where $N_{deg}$ is the number of degrees of freedom of the simulation cell, $N_{deg}=3N_{a}-6$, with $N_{a}$ the number of atoms, *R* is the ideal gas constant, and *T* the absolute temperature measured at time *t*. The shifted potential energy is then divided for $N_{deg}RT$ to compare systems composed of a different number of atoms. The quantity $U^{\prime }\left(t\right)$ used for each trajectory is:

(9) $$U^{\prime }\left(t\right)=\frac{\left[U\left(t\right)-U_{vib}\left(t\right)\right]}{N_{deg}RT}-U^{\prime }\left(0\right),$$

where the dynamic quantities *U* and *T* are computed at each time *t* and time zero is the time at which the external bias starts acting on the system, after 1 ps of equilibration in the absence of the external bias. We define this the shifted and scaled potential energy *U*.

The statistics of 100 trajectories can be used to average over trajectories of $U^{\prime }\left(t\right)$, to further reduce the effects of fluctuations and to extract the information of the reactive event. To perform this task, we average $U^{\prime }\left(t\right)$ over, respectively, reactive and non reactive trajectories. The identification of trajectories as reactive or non reactive is performed by measuring the collective variable used in the pulling experiment (see Equation (2)). The change in CN is so drastic when the chemical reaction occurs that change in CN from the value characterizing the reactant, CN= 2, to that of product, CN=1, is in the fs time range. Therefore, the classification of trajectory is easy.

The identification of reactive and non reactive trajectories allows to compute the fraction of successful trajectories obtaining the product, that is the reactive ratio. This fraction is indicated with $x_{p}$, indicating with the subscript *p* the product. Finally, an approximate estimate of the energy change, in RT units, between product and reactant is given by:

(10) $$\Delta U/RT=\Delta U^{\prime }=x_{p}\langle U_{p}^{\prime }\left(t\right)-U_{r}^{\prime }\left(t\right)\rangle N_{deg},$$

where $x_{p}$ is the product fraction (or reactive ratio) and the average is computed over the time steps where the difference in shifted and scaled *U*, $U^{\prime }$, between product and reactant is stable. The quantity obtained by Equation (10) is substituted in the difference of Equation (8).

The analysis of structural parameters along with time in simulated trajectories was performed with Gromacs (version 2022 [45]) and Plumed (version 2.X [43,46]) codes. Graphical representation and part of the analysis were performed with VMD [32] code.

A hydrogen bond is measured when the geometry of X−H⋯Y atoms fulfill the conditions of d≤ 3.5 Å and α≤ 30°, with *d* the distance between X and Y and α the Y−X−H angle. Potential donors and acceptors are all O and N atoms.

The radial distribution function, g(r), is the probability of distance *r* between two atoms, divided by the same probability in the ideal gas at the same density.

## 5. Conclusions

In this work, we continued the search for a chemical descriptor of nitroacetamide water soluble compounds capable of predicting the anomalous acidity of the C-H group in water. Compound **2** showed a surprisingly high acidity that was attributed to the release of Hα proton to water. To confirm the observation on the basis of a microscopic model, we used an atomistic model in which electron density is described including hundreds of explicit water solvent molecules around reactant and product of the reaction under consideration. The model adequately describes the change in $pK_{a}$ for the two nitroacetamides studied, using as reference the compound with highest $pK_{a}$ (**1**), in agreement with the experimental observation (Table 5). The role of interactions between reactant, product and the liquid environment is confirmed. Thus, the acidity of the compound depends not only on the substituents directly linked to the acidic C-H, but also on more distant substituents in this case placed on the chains of the amide group: it is possible to systematically tune the acidity of nitroacetamides by modifying the hydrophilicity of the substituent on the amide moiety without affecting the sequence O_2_N−CH_2_−C(O)N.

## Figures and Tables

**Table 1:** Titration of **2** at T= 23 °C, concentration 0.0915 M, with NaOH 0.1 M.

mL NaOH	log([HA]/[A])	pH	${\mathrm{pK}}_{a}$
0.3	1.08	3.69	4.77
0.6	0.745	3.95	4.69
0.9	0.5278	4.21	4.74
1.2	0.358	4.40	4.76
1.5	0.210	4.54	4.75
1.8	0.0741	4.70	4.77
2.1	−0.0586	4.86	4.80
2.4	−0.194	5.01	4.82
2.7	−0.340	5.15	4.81
3.0	−0.506	5.35	4.84
3.3	−0.716	5.61	4.89
3.6	−1.0313	6.02	4.99
3.9	−2.0470	6.78	4.73

**Table 2:** Titration of **3** at T= 23 °C, concentration 0.0768 M, with NaOH 0.1 M.

mL NaOH	log([HA]/[A])	pH	${\mathrm{pK}}_{a}$
0.3	1.0013	5.89	6.89
0.6	0.655	6.26	6.91
0.9	0.428	6.50	6.93
1.2	0.245	6.66	6.90
1.5	0.0813	6.83	6.91
1.8	0.0766	6.98	6.90
2.1	0.240	7.15	6.91
2.4	0.422	7.34	6.92
2.7	0.647	7.56	6.91
3.0	0.987	7.89	6.90

**Table 3:** Classification of steered BOMD trajectories using 100 initial configurations for each of the 3 investigated compounds (**1**–**3**). Definitions are explained in Section 4).

Model	Total	Non Reactive	Reactive	Faults	Reactive Ratio ($x_{p}$)
**1**	100	66	5	29	0.07
**2**	100	80	18	2	0.18
**3**	100	85	10	5	0.11

**Table 4:** Potential energy change (kJ/mol) of different chemical species (states) as they are identified within BOMD trajectories (see text for details). Reference energy is that of nitro reactant. The *nitronate* solute energy is corrected for the empirical energy of a proton in water, −1107 kJ/mol.

Compound	*aci*	*enol*	*nitronate*
**1**	60.9	0	−1107
**2**	−9.3	57.3	−621
**3**	29.1	−36.1	−395.2

**Table 5:** Change in $pK_{a}$.

Compound	$\Delta U^{\prime }$	$\Delta pK_{a}$ (Equation (8))	$\Delta pK_{a}$ (Exp.)
**1**	−7.09	0	0
**2**	−11.59	−1.95	−2.47
**3**	−10.04	−1.28	−0.32

## Data Availability

Data are contained within the article and Supplementary Materials.

## Supplementary Materials

The following are available online at https://www.mdpi.com/article/10.3390/molecules29010009/s1, NMR spectra of compound **1** (Figure S1); NMR spectra of compound **3** (Figure S2). Any other data useful to reproduce the results can be requested to authors.
