# Supplementary material for: Probing Reactivity with External Forces: The Case of Nitroacetamides in Water
Source: Molecules. 2023 Dec 19;29(1):9. doi: 10.3390/molecules29010009 (PMC10780269; doi:10.3390/molecules29010009)

Supplementary Materials

Probing Reactivity with External Forces: The Case of Nitroacetamides in Water

Giovanni La Penna and Fabrizio Machetti

Table of Contents

^{1}H and ^{13}C NMR Spectra Figures S1–S2.

Figure S1. ^{1}H NMR (bottom) and ^{13}C NMR (top) spectra in $CDCl_3$ of compound **1**.

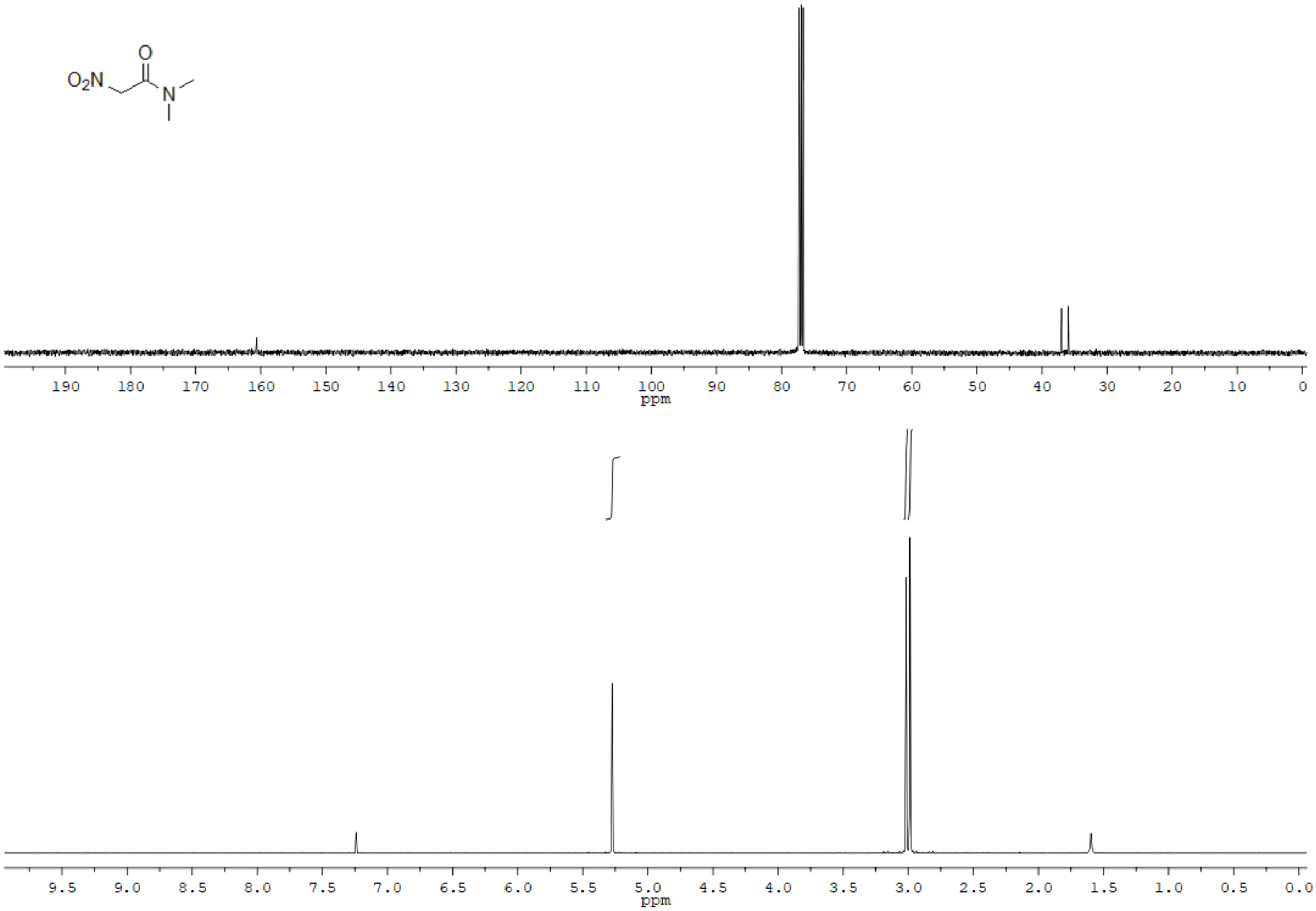

Figure S2. ^{1}H NMR (bottom) and ^{13}C NMR (top with expanded view of CH_2NO_2 carbon) spectra in $CDCl_3$ of compound **3.**

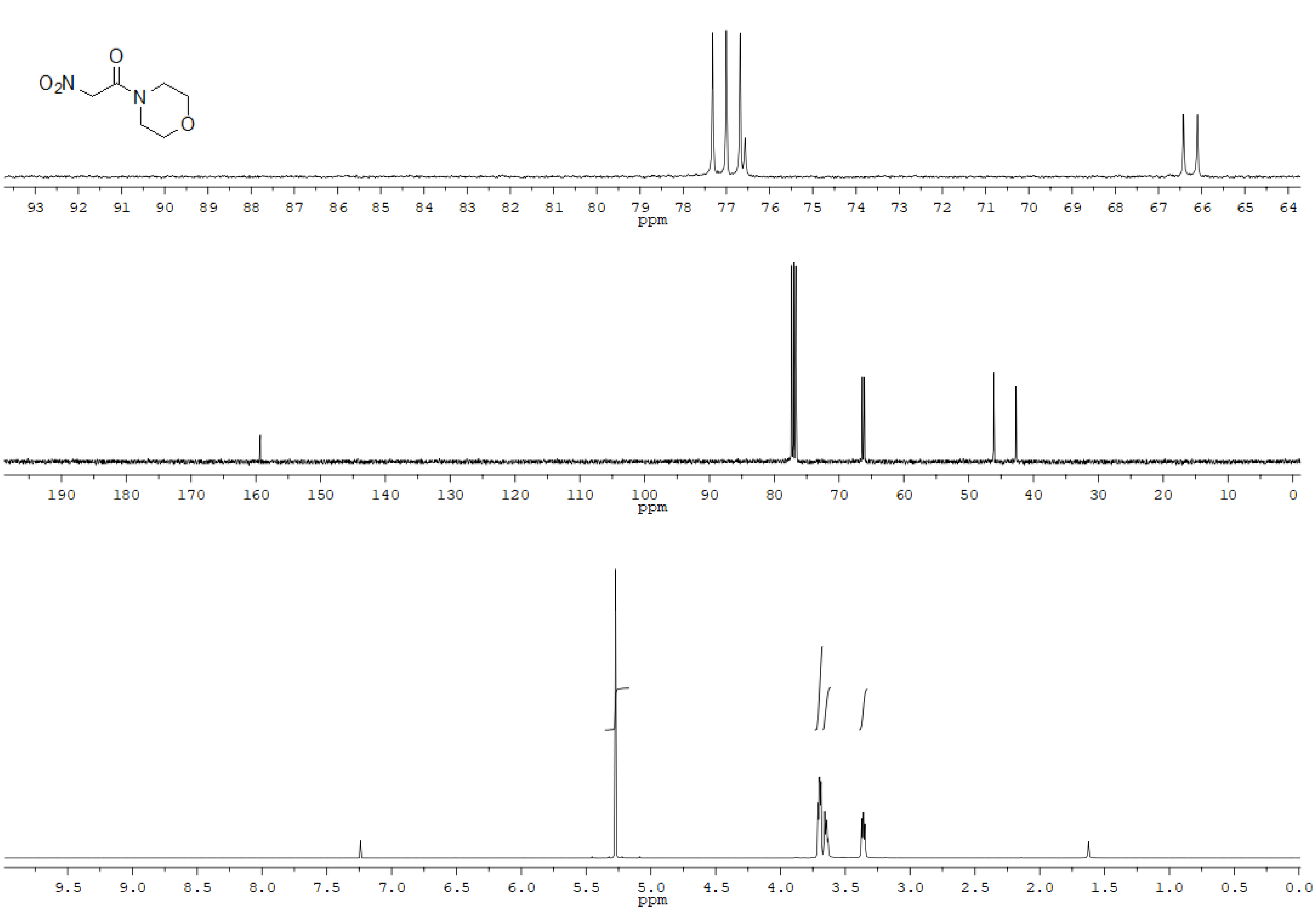

Supplement: Supplementary file 1 [file molecules-29-00009-s001.zip › molecules-2697437-supplementary.pdf]
